# Supplementary material for: Psychotropic medications induced parkinsonism and akathisia in people attending follow-up treatment at Jimma Medical Center, Psychiatry Clinic
Source: PLoS One. 2020 Jul 2;15(7):e0235365. doi: 10.1371/journal.pone.0235365 (PMC7332066; doi:10.1371/journal.pone.0235365)
Supplement: Attachment — Submitted filename: Response to Reviewers.docx [file pone.0235365.s001.docx]

**Manuscript ID:** PONE-D-20-06567

**Title of Manuscript:** Psychotropic medications induced parkinsonism and akathisia in people attending follow-up treatment at Jimma medical center, psychiatry clinic

**Dear Editor,**

We would like to say thank you for assigning reviewer and returning the crucial comments and recommendations. We found the points raised by the reviewer are very interesting to make this article scientifically sound. We addressed all the comments of the editor and reviewer, and we wrote clarifications here under for mutual understanding.

**Editorial comments**: the following points are addressed.

1. **Ethical statement**: As per the editor comment and journal requirement, ethical statement is included in the method section (**P #10, line 180-187**) of the revised manuscript. Accordingly, ethical statement is deleted from the other section.
2. **Funding section**: Thank you for your critical observation and apology for inconsistency in this regard. The funding information that appears in the acknowledgment section is corrected and it is not stated in any part of the revised manuscript. I kindly request to update the funding statement in the online submission form as ***“Financial expense for data collection was covered by Jimma University. The authors have not received financial support for the publication of the article.”***
3. **Language usage, spelling, grammar, and journal formatting style:** As per the recommendation of the reviewer, extensive language revision is made in the current document. In the current revised document through formatting of the entire manuscript is done. A copy of document showing changes (language usage, spelling, grammar, and formatting style) is indicated by track changes and attached as ‘Revised Manuscript with Track Changes.

**Reviewer comments**: Authors of this manuscript are open to accept comments and suggestion, if any, based on the scientific merit and, scope and aim of the journal. We included the response for reviewer # 1 comments and recommendations. To make clear for editors and reviewers, we wrote a response for specific comments and recommendations in the form of table.

**Reviewers’ comments and authors response**

| **Reviewer 1** | |
| --- | --- |
| **Comments and Recommendations** | **Authors Response** |
| *‘Why authors did not include statistical analysis on the correlation of DIP/DIA with specific disorders and/or severity of symptoms of such disorders…..’* | Thank you for the concern and we found this comment very important in lens of clinical implication. The researchers included type of psychiatry diagnosis in the final model of DIA, as there was independent association between the two (**P #20, table 3**). The binary regression between type of psychiatry diagnosis and DIP, found out no statistical significant association between the two. Thus the explanatory variable was removed during multivariable regression analysis and not presented in the table. The discussion point is supplemented in line with the finding and it is added in the last paragraph of discussion section in the revised manuscript (**P #15, line 294-97** ) |
| *‘The concept of use of substance is not properly separated from the DSM definition of substance use disorder and thus the issue of SUD should be addressed’* | Thank you for the comment; we appreciate the critical observation of the reviewer. The researchers used WHO ASSIST screening tool which allows identification of risk level of substance use. We did not engage structured clinical interview with DSM to identify substance use disorder. Thus, the current manuscript is revised to address the risk level rather than focusing simply on substance use; which is the point of concern of the reviewer. In line with the comment of the reviewer we integrate operation definition of each risk level in method section (**P # 9, line 157-59**), result section is modified in **P # 11**, **line 201-06**) and the discussion is revised in **P # 14-15, line 286-93)**. We hope that this increase the scientific sound of the result. |
| *‘What are the columns yes/no in the tables?’* | Thank you once again; we thought that the vague formatting of the tables leads to this question. We formatted the final table (**P #19**) to clearly indicated for what yes/no stands |
| *‘Some precisions on the “physical illness” are needed. Are we including neurological lesions, etc’* | Sure, it is nice point. In this study we only focused on presence of chronic non-communicable disease specifically diabetic and/or hypertension. This is clarified in data collection procedure and instrument sub-section of method part of the document (**P #9, line 164**). Furthermore, patient who had any primary movement disorder and brain lesion was excluded from the study and it is stated in population sub-section of methods and materials (**P #6, line 105-06**) |
| *‘I don’t understand the sentence describing the 4.7% decrease of dopaminergic neuron with aging. This should be made more clear.’* | Sorry for the clarity problem of the statement. The statement is corrected for easy understandability (**PP # 14, line 268-70**). |

**Thank you in advance!**

Shimelis Girma

Corresponding author
